# Ecophysiological and Molecular Analysis of Contrasting Genotypes for Leaf Senescence in Sunflower (*Helianthus annuus* L.) Under Differential Doses of N in Soil

**DOI:** 10.3390/plants13243540

**Published:** 2024-12-18

**Authors:** Daniela E. Becheran, Melanie A. Corzo, Edmundo L. Ploschuk, Salvador Nicosia, Sebastian Moschen, Sofia Bengoa Luoni, Julio Di Rienzo, Nicolas Heinz, Daniel Álvarez, Paula Fernandez

**Affiliations:** 1 Cátedra de Cultivos Industriales, Facultad de Agronomía, Universidad de Buenos Aires, Buenos Aires 1417, Argentina; dbechera@agro.uba.ar (D.E.B.); ploschuk@agro.uba.ar (E.L.P.); 2 Instituto de Biotecnología, UEDD INTA CONICET, Buenos Aires 1686, Argentina; corzo.melanie@inta.gob.ar (M.A.C.); salvalrc@gmail.com (S.N.); 3 Consejo Nacional de Investigaciones Científicas y Técnicas, Famaillá 4132, Argentina; sebamoschen@gmail.com; 4 Laboratory of Genetics, Wageningen University & Research, 6708 PB Wageningen, The Netherlands; bengoaluoni@gmail.com; 5 Facultad de Ciencias Agrarias, Universidad Nacional de Córdoba, Córdoba 5000, Argentina; dirienzojulio@gmail.com; 6 Instituto Nacional de Tecnología Agropecuaria, Estación Experimental Agropecuaria Manfredi, Córdoba 5988, Argentina; heinz.nicolas@inta.gob.ar (N.H.); alvarez.daniel@inta.gob.ar (D.Á.)

**Keywords:** senescence, sunflower, yield, nitrogen

## Abstract

Leaf senescence in plants is the last stage of leaf development and is characterized by a decline in photosynthetic activity, an active degeneration of cellular structures, and the recycling of accumulated nutrients to areas of active growth, such as buds, young leaves, flowers, fruits, and seeds. This process holds economic significance as it can impact yield, influencing the plant’s ability to maintain an active photosynthetic system during prolonged periods, especially during the grain filling stage, which affects plant weight and oil content. It can be associated with different stresses or environmental conditions, manifesting itself widely in the context of climate change and limiting yield, especially in crops of agronomic relevance. In this work, we study the stability of two widely described sunflower (*Helianthus annuus* L.) genotypes belonging to the INTA Breeding Program against differential N conditions, to verify their yield stability in control conditions and under N supply. Two inbred lines were utilized, namely R453 (early senescence) and B481-6 (late senescence), with contrasting nitrogen availability in the soil but sharing the same ontogeny cycle length. It was observed that, starting from R5.5, the B481-6 genotype not only delayed senescence but also exhibited a positive response to increased nitrogen availability in the soil. This response included an increase in intercepted radiation, resulting in a statistically significant enhancement in grain yield. Conversely, the R453 genotype did not show significant differences under varying nitrogen availability and exhibited a tendency to decrease grain yield when nitrogen availability was increased. The response to nitrogen can vary depending on the specific genotype.

## 1. Introduction

Sunflower (*Helianthus annuus* L.) is an economically important crop containing 20–27% proteins and 25–28% edible oil [1]. Sunflower oil is considered to be top quality because it has a high level of polyunsaturated fatty acids [2]. Following rapeseed, mustard, and cotton, sunflower is the fourth major oilseed crop worldwide [3,4,5].

Leaf senescence is a complex mechanism controlled by multiple genetic and environmental variables that influence the yield of crops [6]. A delay or reduction in the senescence rate not only benefits the plant by allowing for a longer photosynthesis time, but can also allow plants to remobilize more nutrients accumulated in the senescence leaves to maximize their final contribution to plant reproduction [7]. In plants, senescence is a prelude to cell (organ) death, and, during this process, metabolites and macromolecules released are reutilized for plant growth [8]. The advances in genomic and post-genomic technologies in the last decade allowed the discovery and functional characterization of many genes simultaneously. Functional genomics allows the integrated analysis of data derived from transcriptomics and other post-genomic disciplines, such as proteomics and metabolomics, leading to the identification of the function and regulation of genes in certain biological processes. The scenario of genomic biotechnology has radically changed the context of application of classical statistical techniques and is leading to a paradigm shift in the analysis of biological data.

The implementation of RNA-seq technology for the study of leaf senescence in sunflower plants has been recently studied by our group. In this study, we identify gene expression profiling, metabolic pathways and gene coexpression networks associated with leaf senescence in contrasting sunflower genotypes [9].

The response to nitrogen fertilization in sunflower (and its complementation with P) regarding yield and grain quality is strongly related to the soil supply, the genotypic characteristics, and an adequate water supply during the crop cycle [10]. Furthermore, it is necessary to consider that the evaporative demand by the crop is 300 mm, as well as the high water and N requirements during the period ranging from 2–4 leaf pairs to anthesis (approximately 20 to 80 days after the emergence), where 75% of the N absorption is produced in the 30 days around anthesis [11]. This is a key factor, since nitrogen plays an important role in the processes of growth, development, leaf senescence, and yield generation [12]. If the soil is provided with adequate amounts of N, this supply will be sufficient to meet the demand of the crop and, therefore, fertilization will be an unnecessary practice, giving a situation of luxury consumption by the plant. Early applications of nitrogen [13] generate a greater absorption of nitrogen by the sunflower. Hence, crop yields depend directly on the duration of the foliar area between the stages of anthesis and physiological maturity [14]. An important parameter to consider is the NUE (nitrogen use efficiency), which indicates the crop’s capacity to incorporate nitrogen from soil sources (acquisition) and utilize it for growth (utilization). However, both the response to fertilization and the NUE have high genetic variability and environmental responses that may influence these parameters [10]. In turn, it is necessary to consider how the nitrogen supply affects the plant’s capacity to capture radiation and convert it to dry weight. Previous studies showed that this capacity mainly depends on two components: (I) the green leaf area of the crop capable of capturing the light which can be potentially limited by leaf duration, which is related, in turn, to leaf senescence [15]; (II) the photosynthetic activity per unit of green surface of the crop (the capacity to convert intercepted radiation into assimilated, that is, chemical energy), which is related to the accumulation of nitrogen in leaves [16].

The signal of senescence occurs in response to a combination of environmental stimuli and gene expression. The leaf is the main photosynthetic organ of plants, and its development is a complex process governed by a combination of developmental and environmental stimuli which also involves post-transcriptional modifications due to the interference of the environment in the gene expression.

During early development, the leaf is a sink that receives nutrients from the rest of the plant; however, as soon as it reaches full photosynthetic capacity, it becomes the main source organ of the plant [17].

Under nutritional deficiency, where senescence is prematurely triggered, the increase in soluble sugars can lead to a loss in both functional and structural integrity in cell membranes, increasing the lipid catabolism of the membrane and the production of sugar by gluconeogenesis [18]. During senescent leaf metabolism, the assimilation of carbon and nitrogen is replaced by a catabolism of chlorophyll and macromolecules, such as proteins, RNA, and membrane lipids, whose degradation marks the phase of senescence [19]. This event should have prevalence in the advanced grain filling phase, driving the remobilization of the nutrients stored in the leaves and in the stem, which in optimal conditions will satisfy the demand of the destination.

The “stay green” genotype (SG) condition is a mechanism to mitigate the premature leaf senescence and potentially to extend the period of PAR (photosynthetically active radiation) capture by a crop, promoting a delay in the activation of the signaling cascade of nutrient remobilization and foliar senescence. This impacts several parameters. Firstly. if the stay green genotype is functional and the photosynthetic machinery does not lose efficiency, the RUE (radiation use efficiency) increases, leading to a greater accumulation of photoassimilates. On the other hand, due to the hormonal balance (particularly an increase in the cytokinin concentration), many photoassimilates may end up in the roots, generating a more active root system that will be able to absorb more available nutrients, a situation that will have an impact on the delay of the remodeling of the nutrients (balance of C/N) [20]. These two phenomena (the remobilization and the contribution of edaphic N) have equal preponderance in the demand of N by the reproductive destinies, which is the reason why both are an important source of nutrients. Additionally, the primary determinant of crop biomass production is cumulative net photosynthesis over the growing season [21], where photosynthesis is defined as a plant process using the energy from light to convert carbon dioxide (CO_2_) and water (H_2_O) into oxygen (O_2_) and carbohydrates [22]. The aim of this work is to characterize two sunflower genotypes that differ in leaf senescence under different soil nitrogen levels. We aim to evaluate the stability of these genotypes under control and nitrogen-supplemented conditions to validate the treatment independence for the age-induced senescence trait under different nutritional environments/conditions.

## 2. Results

### 2.1. Environmental Conditions and Phenology

**Figure 1:**
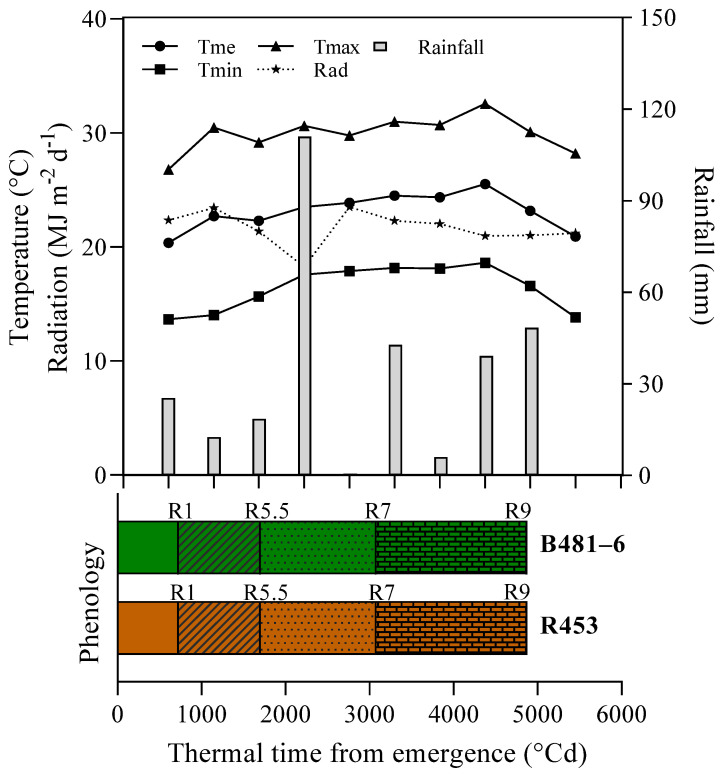
Meteorological conditions during the crop cycle. Values are daily means of medium (Tm), maximum (Tmax), and minimum (Tmin) temperature and daily photosynthetic incident radiation (Rad) and accumulated rainfall (Rainfall). Bars indicate the phenology of the B481-6 and R453 genotypes. R1 corresponds to the “star visible” stage, while R5.5 marks mid-anthesis, R7 indicates when the rear of the head initiates a transformation to a pale-yellow hue, and, at R9, the bracts become yellow and brown, following the scale proposed by Schneiter and Miller (1981) [23].

The phenological study revealed a precise sequence of events from the seeding stage to physiological maturity, providing a detailed insight into plant development. The initial phase (from seedling to emergence) lasted only four days, highlighting the rapid germination and emergence of sunflower seedlings in our study environment. Following emergence, the time to R1 stage (characterized by floral bud formation) was 727 °Cd for both the B481-6 genotype and R453 genotype. The time to R5.5 and physiological maturity was similar for both genotypes, completing a total phenological cycle of 1795 °Cd (Figure 1).

In relation to the climatic conditions, Figure 1 displays the average values of temperature, radiation, and accumulated precipitation. The average temperature remained around 24 °C during the flowering period, with recorded minimums between 18 °C and a maximum of 32 °C. Regarding radiation, it remained constant at approximately 21 MJ m-2d-1 during the same stage.

Precipitation was consistent, although the highest accumulation was observed in the region R1 stage, reaching 111 mm, and around R5.5, with 43 mm.

### 2.2. Net Photosynthesis and Stomatal Conductance

**Figure 2:**
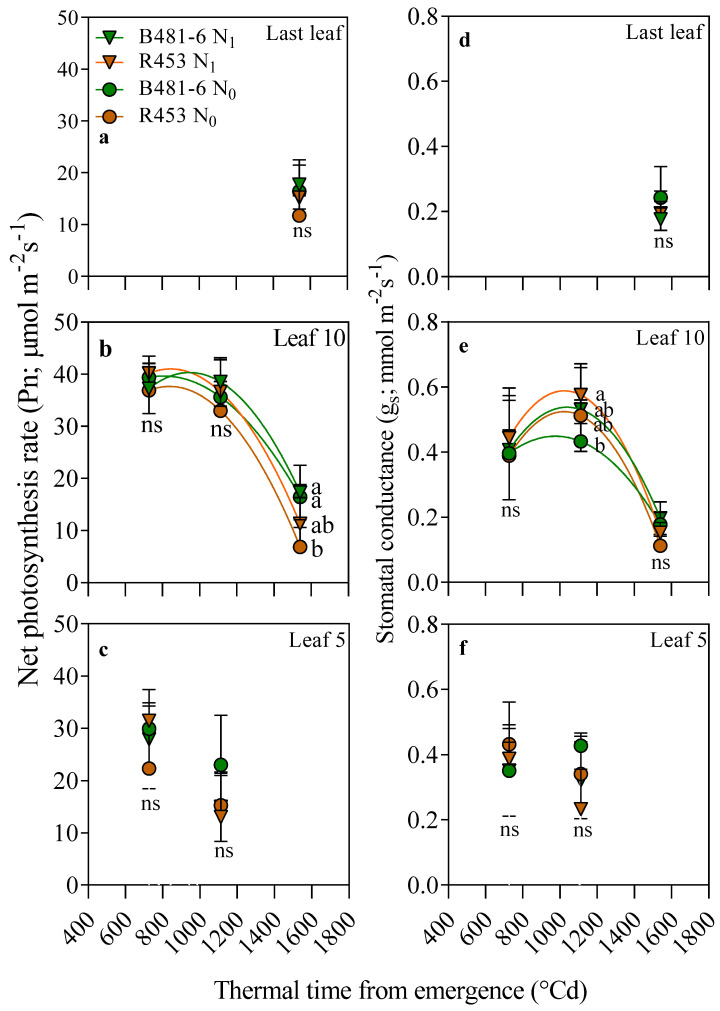
The net photosynthesis (Pn) and stomatal conductance (gs) data for the 5th leaf (**c**,**f**), 10th leaf (**b**,**e**), and last leaf (**a**,**d**) in genotypes B481-6 and R453 with different soil nitrogen levels as a function of thermal time to emergence (°Cd). R1 corresponds to observations taken at 727 °Cd, R5.5 corresponds to observations taken at 1112 °Cd, and R8 corresponds to observations taken at 1540 °Cd, following the scale proposed by Schneiter and Miller (1981) [23]. Vertical bars indicate ± standard error. Different letters at each phenological stage indicate significant differences for Tukey’s test at *p* = 0.05.

No differences among treatments and genotypes were observed for net photosynthesis (Pn) and its related traits at leaf 5 and the last leaf (Figure 2). At leaf 10, Pn remained stable (around 35 µmol m^−2^ s^−1^ across all treatments) during both the R1 and R5.5 stages. On average, a drastic drop was observed at the R8 stage, although this decline was more pronounced in the early-senescence genotype R453 (dropping to approximately 5 µmol m^−2^ s^−1^ in the unfertilized treatment, Figure 2b) compared to the delayed-senescence genotype B481-6. Interestingly, the Pn pattern observed at R8 was not mirrored by stomatal conductance (gs), as no differences among treatments were detected for this trait at the same thermal time (Figure 2e). However, at the R5.5 stage, gs was 30% lower in the fertilized genotype B481-6 than in the unfertilized early-senescence one, despite no differences being observed for Pn.

**Figure 3:**
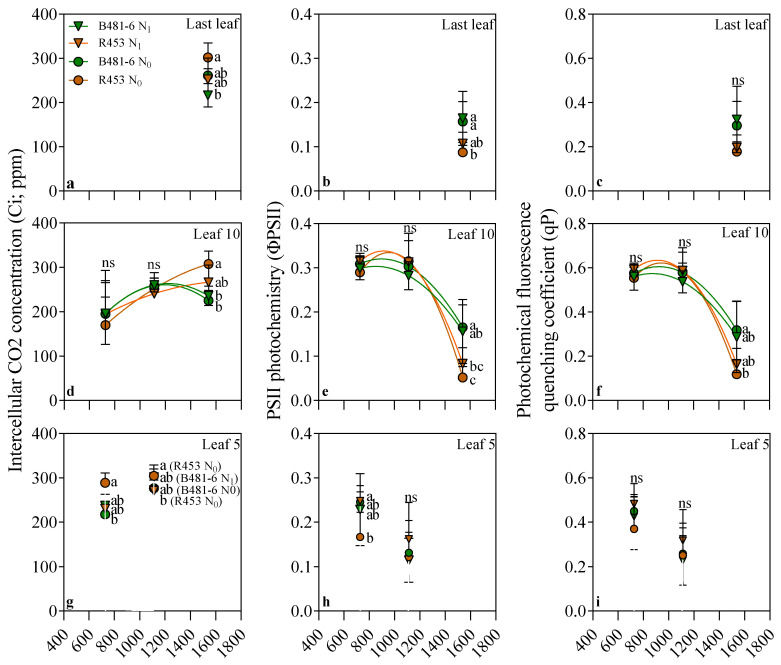
Intercellular CO_2_ concentration (Ci), quantum yield of PSII photochemistry (ΦPSII), and photochemical fluorescence quenching coefficient (qP) data for the 5th leaf (**d**–**f**), 10th leaf (**g**–**i**), and last leaf (**a**–**c**) in genotypes B481-6 and R453 with different soil nitrogen levels as a function of thermal time to emergence (°Cd). R1 corresponds to observations taken at 727 °Cd, R5.5 at 1112 °Cd and R8 corresponds to observations taken at 1540 °Cd, following the scale proposed by Schneiter and Miller (1981) [23]. Vertical bars indicate ± standard error. Different letters at each phenological stage indicate significant differences for Tukey’s test at *p* = 0.05.

In contrast to the trends observed for Pn at leaf 10, no clear temporal tendences were found for intercellular CO_2_ concentration (Ci, Figure 3d). However, at the R8 stage, the results showed a clear association with Pn: the higher the Pn, the lower the Ci. Consequently, Ci was reduced by up to 30% (from 300 to 200 ppm) in the delayed-senescence genotype compared to the early-senescence one, independent of N availability. Interestingly, this trend was also observed in the last leaf (Figure 2a) and at leaf 5 (Figure 2f). Even more notable is the fact that the fluorescence parameters ΦPSII and qP followed the same pattern as Pn in leaf 10 (Figure 3e,f). Both traits remained stable until stage R5.5, at around 0.3 and 0.6 for ΦPSII and qP, respectively, but sharply decreased by the R8 stage. However, the drop was more pronounced in the early-senescence R453 genotype, reaching approximately 0.5 and 0.1 for ΦPSII and qP, respectively.

### 2.3. Green Leaf Area and Intercepted Radiation

**Figure 4:**
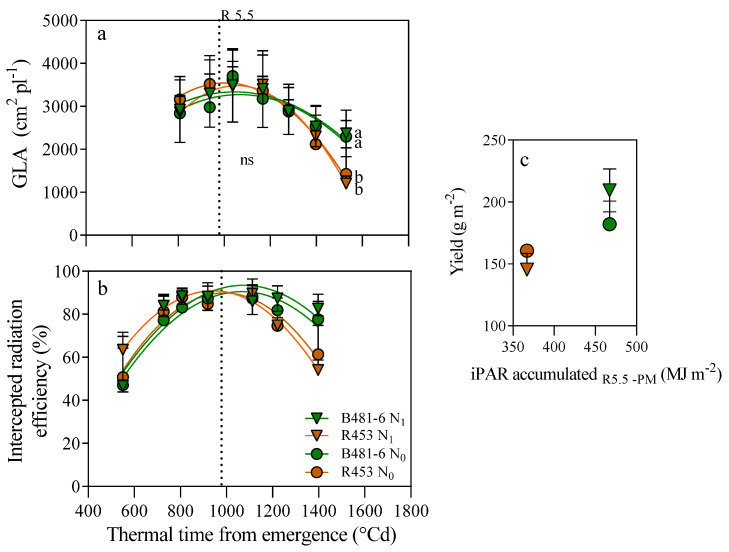
The green leaf area (GLA) per plant (**a**) and fraction of intercepted PAR (iPAR %) (**b**) as a function of thermal time to emergence (°Cd). Insets (**c**): Yield (g m^−2^) as a function of intercepted PAR accumulated during the period from R5.5 to physiological maturity (R5.5-PM; MJ m^−2^) in genotypes B481-6 and R453 with different soil nitrogen levels. A dashed line indicates mid-anthesis (R5.5). Symbols indicate nitrogen availability and colors indicate line. Vertical bars indicate ± standard error. Different letters at each phenological stages indicate significant differences for Tukey’s test at *p* = 0.05.

A similar trend to that observed for Pn was noted in the green leaf area (GLA, Figure 4a). At leaf 10, no differences were detected among treatments or genotypes from R1 until 1400 °Cd (around the R7 stage). Once again, the decrease in GLA was more pronounced in the R453 genotype by the R8 stage compared to B481-6, with no significant differences between fertilized and non-fertilized plants. At this stage, a significant reduction of 50% was observed in R453 and 20% in B481-6 (*p* < 0.05). Additionally, the efficiency of intercepted radiation (%) closely correlated with the trends in GLA (Figure 4b). The highest values for intercepted radiation efficiency were recorded at the R5.5 stage, followed by a decrease of 35% in R453 and 8% in B481-6, irrespective of the different nitrogen levels in the soil.

### 2.4. Yield and Biomass

**Figure 5:**
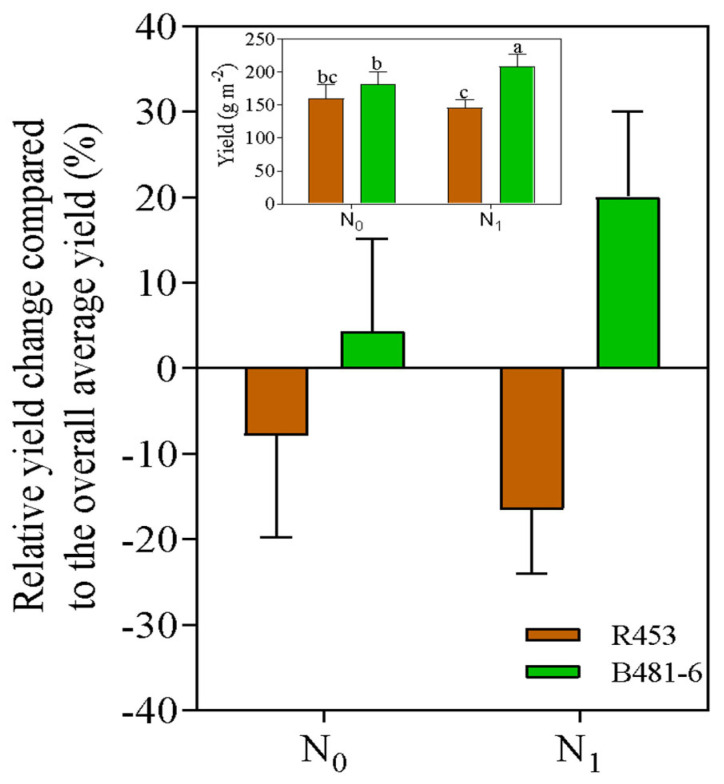
Relative yield changes compared to the overall average yield (%) for the entire experiment under the unfertilized condition (N0) and the fertilized condition (N1) in lines R453 and B481-6. Insets: Yield (g m^−2^) as a function of fertilized conditions. Different letters indicate significant differences (*p* < 0.05) between treatments.

In contrast to the previously described traits, a strong interaction between genotype and nitrogen level was detected for yield. No significant differences were observed between genotypes under the N0 treatment. However, under fertilized conditions, the delayed-senescence B481-6 produced 2000 kg ha^−1^, while the yield of R453 was 25% lower (Figure 5). Additionally, a positive association was found between intercepted PAR (iPAR) and yield during grain filling, with a more pronounced response in B481-6 compared to R453 under the same conditions (Figure 4c).

Surprisingly, the total aerial biomass weight per plant was the same at both the R5.5 and R8 stages for the overall treatments and genotypes (Table 1). Main stem weight followed a similar pattern, while leaf weight per plant was significantly higher in the fertilized B481-6 genotype.

### 2.5. Molecular Analysis

Through qPCR assays, we analyzed the expression profiles of the candidate gene HaNAC01, a transcription factor, at four different time points (stages R1, R3, R7, and R8) for two sunflower genotypes. In a previous study, the HaNAC01 transcription factor was validated as a SAG (senescence-associated gene) in sunflower [15,16].

**Figure 6:**
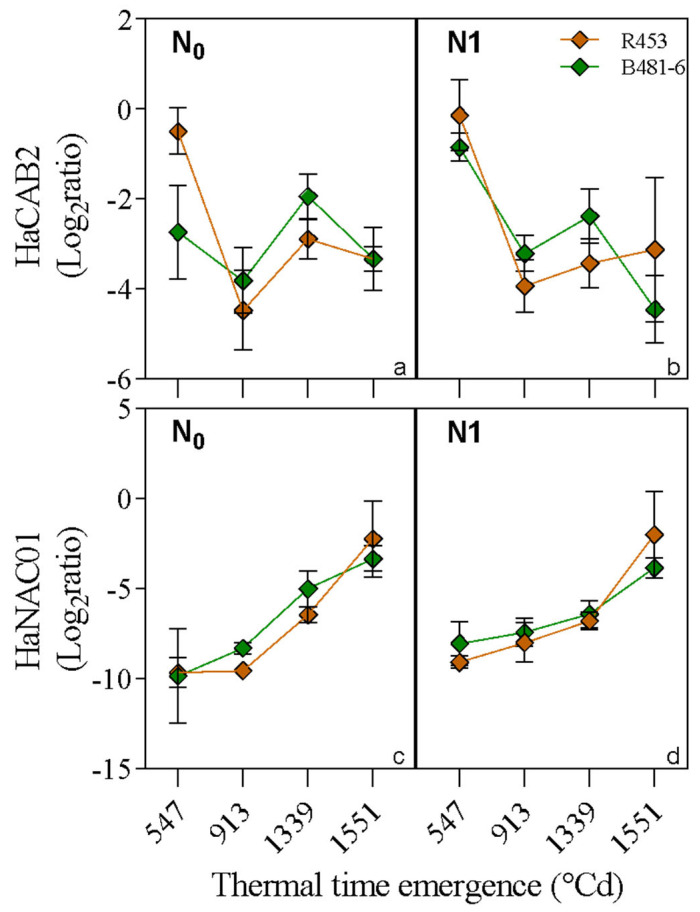
qPCR Assays: Relative expression profiles of transcription factors under unfertilized (N0) and fertilized (N1) conditions in genotypes R453 and B481-6. (**a**,**b**) HaCAB2 (senescence marker gene; SDG in sunflower) and (**c**,**d**) HaNAC01—SAGs in sunflower. Relative transcript levels are depicted as the ratio (log2 scale) between the gene expression of the premature senescence genotype and its contrasting delayed senescence genotype.

Genotype R453 exhibited slightly lower relative expression levels compared to B481-6 for the HANAC01 transcription factor, with a significant increase in expression during maturity (Figure 6c,d). In the unfertilized condition (N0), lower levels of HaNAC01 were observed compared to the fertilized condition (N1), but, at maturity, the relative expression levels increased to similar values. These results are like those of a previous study [24], in which NAC FTs were overexpressed in the prematurely senescing genotype.

The sunflower HaCAB02 gene presents high sequence similarity to a chlorophyll A/B-binding protein 2 [25]. This gene is an indicator of advanced senescence phases and, consequently, differences in expression patterns between contrasting genotypes might arise in later sampling times [26]. The relative expression levels of HaCAB2 between contrasting genotypes showed slight differences between fertilized and unfertilized conditions (Figure 6a,b). These differences changed as senescence progressed, and where expression levels decreased, with the R453 genotype showing a more pronounced decrease. At maturity, relative expression levels remained constant in the unfertilized condition, while in the fertilized condition, the relative expression level of HaCAB2 was higher in the R453 genotype and lower in the B481-6 genotype.

## 3. Discussion

Detailed research on the physiology and performance of sunflower (*Helianthus annuus* L.) in relation to soil nitrogen availability has provided valuable insights into the underlying mechanisms of variability in the yield and physiological response of different genotypes [15,19]. In various crops, such as sunflower, delaying leaf senescence can have a significant impact on grain production by maintaining photosynthetic leaf surface during the reproductive phase [27,28]. However, this process is complex and is influenced by various temporal, spatial, biotic, and abiotic stresses.

The study conducted on inbred lines of sunflower revealed interesting patterns in plant response to soil nitrogen availability. Significant variations were observed in physiological aspects, such as net photosynthesis (Pn), at the end of the ontogenetic cycle (R8, Figure 2), influenced mainly by genotype and not by nitrogen treatment. This increase in photosynthesis was related to a higher green leaf area per plant and percentage of intercepted radiation (Figure 4), as previous research has demonstrated that the larger nitrogen availability of the delayed-senescence genotype can lead to increases in those traits [28,29,30]. Notably, no association was found between Pn and stomatal conductance (gs, Figure 3), discarding possible causes related to diffusion processes, although the antecedents about the effect of nitrogen availability over the latter are contrasting. Thus, low gs rates have been evidenced in plants growing with low nitrogen availability [31,32,33] as well as high [34] or null [34,35] availability.

Independently of stomata conductance, lower Ci concentrations were found for the delayed-senescence B481-6 genotype, when Pn rates are higher than those for the R-453. This means that the photosynthesis enhancement was produced by other no-stomata limiting processes (instead of gs), such as the biochemical pathway. Indeed, the lower ΦPSII and qP associated with the higher Pn is explained by a possible lack of Rubisco enough to support the photosynthesis activity [36].

Despite the observations for photosynthesis and related traits at the R8 stage, a strong interaction between genotype and nitrogen level was detected for yield, as the advantage of the delayed-senescence B481-6 genotype was clearly exacerbated under fertilized conditions (Figure 5). Interestingly, no differences in total aerial biomass were found between treatments and genotypes, suggesting that the proportion of total biomass allocated to the reproductive fraction was increased. Although the underlying causes remain unclear, one possible explanation is that under the N1 treatment, a higher growth rate may have led to a greater integral of the generative area, which is crucial for determining potential yield [37]. Genotypes with delayed senescence, such as B481-6, may have capitalized on this potential by maintaining higher active photosynthesis during critical stages of achene filling, particularly in conditions where this process relies more on post-anthesis photosynthesis than on stored reserves. The greater leaf area and higher percentage of intercepted radiation would have been key to sustaining a high net photosynthesis rate (Pn) during the reproductive phase, providing carbohydrates for achene filling. Furthermore, the fact that higher Pn rates were not associated with stomatal conductance (gs) but with optimized biochemical processes suggests that the delayed-senescence genotype is leveraging internal metabolic pathways to maintain photosynthetic activity even during the final stages of reproductive maturity. Thus, the B481-6 genotype may have an effectively reduced senescence rate, optimizing resource allocation to reproductive structures while sustaining photosynthetic activity. These results suggest that genotypes with delayed senescence can take advantage of a longer leaf area duration to improve yield without compromising vegetative and reproductive functions. Further research is needed in order to check this hypothesis.

Additionally, gene expression analysis revealed interesting differences among genotypes, especially concerning nitrogen availability. The expression levels of HaCAB2 showed significant variations among genotypes and fertilization conditions, suggesting a differential response to nutrient availability, particularly nitrogen. These results highlight the complexity of the mechanisms regulating plant responses to nutritional stress and the importance of considering the specific growth stage when evaluating the effects of fertilization on gene expression.

Overall, these findings provide a deeper understanding of the interaction between sunflower and soil nitrogen availability, with important implications for genetic improvement and sustainable agricultural practice. However, further research is needed to fully elucidate the underlying mechanisms of these responses and their practical application in agriculture.

In previous work [6,15,24,38] it was identified that the downregulation of the CAB gene over time is lower when senescence progresses, due to this gene being a chlorophyll a/b binding protein, and, therefore, highly dependent on net photosynthesis. These results indicate that genotype B481-6 could be classified as a putative stable functional stay green genotype, and the differences in time-to-senescence rate are genetically dependent rather than due to differential photosynthesis due to differences in nitrogen. Here, we observed this differential expression, both because of the genotype and the effect of the N1 treatment. This is why, as with other types of programmed cell death, plant senescence is accompanied by a decrease in protein synthesis (e.g., ribulose 1,5 bisphosphate carboxylase/oxygenase (Rubisco)], expression of senescence-downregulated genes (SDGs) often related to photosynthesis (e.g., *CAB* gene encoding a chlorophyll a/b-binding protein), and upregulation of senescence-associated genes (SAGs), as well as de novo synthesis of proteins [18], being one of the most suitable molecular indicators of senescence progression [17,25].

In this study, a novel sunflower NAC transcription factor, *HaNAC01*, previously identified by our group [24] was evaluated, and it displayed increased expression during leaf development as a positive regulator of leaf senescence. Similar results were reported in Arabidopsis [39,40]. Regarding *HaNAC01* [6,26] a putative orthologous TF for sunflower of *ORE1* in Arabidopsis [41,42], it also presents a genotype-dependent expression. This TF advanced its expression as thermal time progressed, and, consequently, natural senescence began in the plant. This effect could be seen in Arabidopsis, wheat, soybeans, and petunia [43,44,45]. NAC genes that are negative regulators of senescence appear to be rare: only Arabidopsis *JUB1 gene* was identified as a negative regulator of senescence [44]. Additionally, three NAC genes were identified in barley (*HvNAC004*, *HvNAC042*, and *HvNAC046*) with decreased expression at late senescence stages [45].

This is the first work which characterizes and evaluates two contrasting sunflower genotypes not only under varying soil nitrogen supplements for age-induced leaf senescence trait, but also for senescence-associated transcription factors associated with the process. The demonstrated stability of these genotypes across control and nitrogen-supplemented conditions validates them as future candidates for a biparental population in a regional breeding program.

## 4. Materials and Methods

### 4.1. Plant Material and Experimental Conditions

The experiment was conducted at the Instituto Nacional de Tecnologia Agropecuaria (INTA) Biotechnology Institute during the 2019–2020 growing season under field conditions. Previously selected inbred lines, namely R453 (early-senescence) and B481–6 (delayed-senescence) genotypes, belonging to the INTA Sunflower Breeding Program and INTA Sunflower Germplasm Collection in Manfredi, were used.

Sowing took place on 17 November 2019, in seedling trays inside a greenhouse, where the plants grew until reaching the two true-leaf stage with a length of more than 4 cm (V4, according to [23]). Subsequently, the plants were acclimated outdoors before being transplanted to their final location. Prior to sowing, soil sampling was conducted in the upper 0.40 m layer to determine nitrogen availability. The plot average initial nitrogen levels were 72 kg ha^−1^.

The plots consisted of 10 rows with a spacing of 50 cm and a length of 5 m. The experiment was conducted using a split-plot design with a block structure. The main plot treatment involved nitrogen fertilization with two specific conditions: control (no fertilization, denoted as N0) and fertilized with the application of 100 kg of nitrogen (N) per hectare in the form of granular urea (denoted as N1). This fertilization condition was applied in bands at the V6 phenological stage [23] and increased the initial nitrogen level mentioned above to a value of 200 kg ha^−1^. The sub-plot treatment corresponded to the evaluated genotypes (R453 and B481-6). This design comprised 2 blocks of 50 m^2^, each with 2 sub-plots of 25 m^2^. The spatial arrangement was set with a row spacing of 0.5 m and a plant spacing of 0.25 m, resulting in a density of 8 plants per unit area.

Time was expressed on a thermal time basis by daily integration of air temperature with a threshold temperature of 6 °C and with plant emergence as thermal time origin [46].

Chemical control for weeds, pests, and diseases was implemented as needed, following local agronomic practices. Rainfall throughout the crop cycle was supplemented with a drip irrigation system.

### 4.2. Measurements

#### 4.2.1. Phenology

Crop phenology was registered following the scale proposed by Schneiter and Miller (1981) [23] to distinguish different phenological stages. For instance, the R1 stage (denoted as “visible star”) means the point at which the inflorescence, encircled by immature bracts, becomes apparent at the apex of the plant. Additionally, at the R5.5 stage, occurring during mid-anthesis, approximately 50% of the plants have reached this critical point.

Subsequent stages, namely R7 and R8, mark significant developments in the crop’s maturation process. At R7, the rear of the head initiates a transformation to a pale-yellow hue, and the grain attains 50% of its final dry weight. Advancing to R8, the back of the head takes on a yellowish tint, although the bracts persist in their green state, and the grain achieves 90% of its ultimate dry weight. Phase duration was expressed on a thermal time basis by daily integration of air temperature with a base temperature of 6 °C and with plant emergence as thermal time origin [46].

#### 4.2.2. Climate Conditions

Hourly values of incident solar radiation, air temperature, and daily rainfall were recorded using a meteorological dataset at the Castelar Experimental Station of the National Institute of Agricultural Technology (INTA) at the experimental site. Incident photosynthetically active radiation (IPAR) was assumed to represent 45% of the incident solar radiation [47].

#### 4.2.3. Radiation Interception

From the R1 stage to physiological maturity (PM), photosynthetically active radiation interception (iPAR) of the canopy was determined with a linear ceptometer (CAVARAD, Cavadevises, Argentina) between 12:00 PM and 02:00 PM on clear days. Measurements were made at each plot, one above the canopy, to determine the incident PAR (I0), and another below, following the senescence profile, representing transmitted PAR (It). The fraction of intercepted PAR (iPAR %) was calculated as (I0 -It)/I0. Photosynthetically active radiation intercepted by the crop (iPAR; MJ m^−2^ day^−1^) was calculated each day as the product of iPAR %, incident global radiation (MJ m^−2^ day^−1^) and 0.48 (i.e., ratio of photosynthetically active to total radiation [48].

#### 4.2.4. Green Leaf Area (GLA)

The evolution of the green leaf area was assessed through periodic measurements of the maximum width of each leaf in selected plants representative of the plots. The degree of greenness was visually evaluated by a single observer, through a comparison of the ratio between the green and yellow sections on each leaf (ranging from 100% to 0%). The subsequent determination was made by calculating the green leaf area (GLA) per plant [23,49]:

$$\mathrm{GLA}\;({\mathrm{cm}}^{2})=\frac{\left(\mathrm{Leaf}\;\mathrm{area}\;\left({\mathrm{cm}}^{2}\right)\times \;\mathrm{Percentage}\;\mathrm{of}\;\mathrm{greenness}\right)}{100}$$

where the leaf area (cm^2^) = 1.528 × (leaf width) 1.7235

#### 4.2.5. Dry Weight, Yield and Its Components

At the R5.5 and R8 stages and physiological maturity, total aboveground biomass was harvested from 8 plants in each plot and separated into stem, leaves, and head. The material was dried in oven at 65 °C until it reached a constant weight, and the dry weight was measured. In addition, in the physiological maturity stage, the sunflower heads were collected from a total of 10 plants. The number and weight of seeds were then measured for each collected head. To assess the performance by genotype, the following formula was used [23,48]:

$$\mathrm{Yield}\;\left(g\right)=\frac{\mathrm{Average}\;\mathrm{fresh}\;\mathrm{weight}\;\mathrm{of}\;\mathrm{seeds}\;\mathrm{per}\;\mathrm{plant}\;\left(g\right)}{\mathrm{Number}\;\mathrm{of}\;\mathrm{seeds}}\times 1.000$$

#### 4.2.6. Leaf Physiological Measurements

Net photosynthesis rates (Pn), stomatal conductance (gs), intercellular CO_2_ concentration (Ci), quantum yield of PSII photochemistry (ΦPSII), and photochemical fluorescence quenching coefficient (qP) were determined for all treatments at approximately the R1 and R5.5 stages on the 5th and 10th fully expanded leaves of the plants, as well as at the R8 stage on the 10th and last fully expanded leaves (corresponding to the 15th fully expanded leaf). Measurements were taken from the lower, middle, and upper strata of the plants, assessing the 5th leaf (lower stratum), the 10th leaf (middle stratum), and the last leaf (upper stratum). A portable infrared gas analyzer (IRGA), specifically the Li-Cor 6400 from Li-Cor Inc. (Lincoln, NE, USA), was used for the measurements under 2000 µmol m^−2^ s^−1^ PPFD. Saturating light was provided by the 6400-40 leaf chamber fluorometer, using a mixture of 80% red and 20% blue light. Airflow, CO_2_ concentration in the reference chamber, and block temperature were automatically controlled by the equipment at 300 µmol s^−1^, 400 µmol mol^−1^ (ppm), and 25 °C, respectively.

#### 4.2.7. Quantitative RT-PCR Analysis

At four different time points (stages R1, R3, R7, and R8), the tenth leaf (numbered for the bottom to the top of the plant) of three plants per genotype under two conditions of nitrogen were harvested, frozen in liquid nitrogen, and stored at −80 °C until use. High-quality total RNA was isolated from 100 mg of tissue using TRIZOL and following the manufacturer’s instructions. Genomic DNA was eliminated using DNase I (Invitrogen, Buenos Aires, Argentina). RNA concentration was quantified by a Nanodrop. The purity of total RNA was determined by a 260/280 nm ratio, while the integrity was assessed by electrophoresis in 1.5% (*w*/*v*) agarose gel.

A quantitative analysis of RT-PCR was performed, as described by López Gialdi et al. [27], to examine the expression of genes associated with leaf senescence. RNA treated with DNase was used for reverse transcription using the Superscript III kit (Invitrogen) and random hexamer primers. qPCR reactions were conducted with specific primers and the FastStart Universal SYBR Green Master (Rox) in a thermocycler, incorporating negative controls and verifying amplicon specificity through melting curve analysis. The thermal profile was set to 95 °C for 10 min, with 40 cycles of 95 °C for 15 s and the hybridization temperature for 1 min. The optimization of the hybridization temperature for each primer was previously tuned up. Each condition was biologically and technically replicated three times. The relative expression of senescence-related genes, namely HaNAC01 and HaCAB2, was estimated using the gene EF-1α and actin as reference genes, which were previously selected as references genes [19]. Amplification efficiencies and Ct values were determined for each gene and condition using the LinRegPCR Analysis of Quantitative RT-PCR Data software v 11.0 [50]. The expression profiles were compared between early and late senescence genotypes by using specialized software.

### 4.3. Data Analysis

Statistical analysis included a study of the differences between treatments using the InfoStat Professional v.1.1 software [51]. This software allowed for tests of variance using a split-plot ANOVA with “nitrogen” as the main factor and “genotype” as the minor factor. To compare treatment means, the Fisher LSD (least significant difference) test was employed at a significance level of 0.05. Additionally, GraphPad Prism 5 for Windows (GraphPad Software, San Diego, CA, USA, www.graphpad.com) was used to generate graphs.

## Figures and Tables

**Table 1:** Flower head (g pl^−1^), leaves (g pl^−1^), stem (g pl^−1^), and total aerial biomass (g pl^−1^) of two sunflower inbred lines grown under different nitrogen conditions: unfertilized condition (N0) and fertilized condition (N1) at the R5.5 and R8R7 stages. Mean values and significance for the main factors (G = genotypes; N = nitrogen) and interactions are presented.

Phenological Stages	Nitrogen	Genotypes	Flower Head (g pl^−1^)	Leaves (g pl^−1^)	Stem (g pl^−1^)	Total Aerial Biomass (g pl^−1^)
R5.5 stage	N_0_	B481-6	95.7 ^a^	134.0 ^a^	256.3 ^a^	486.0 ^a^
		R453	171.3 ^a^	158.6 ^a^	376.5 ^a^	706.4 ^a^
	N_1_	B481-6	162.0 ^a^	204.0 ^a^	437.3 ^a^	803.3 ^a^
		R453	143.4 ^a^	169.9 ^a^	275.7 ^a^	710.3 ^a^
		G	ns	ns	ns	ns
		N	ns	ns	ns	ns
		GxN	*	ns	ns	ns
R8 stage	N_0_	B481-6	334.3 ^a^	140.1 ^b^	299.1 ^a^	773.5 ^a^
		R453	406.9 ^a^	133.6 ^b^	296.9 ^a^	837.4 ^a^
	N_1_	B481-6	439.7 ^a^	181.4 ^a^	313.7 ^a^	934.8 ^a^
		R453	496.1 ^a^	123.4 ^b^	305.3 ^a^	924.7 ^a^
		G	ns	**	ns	ns
		N	ns	ns	ns	ns
		GxN	ns	*	ns	ns

ns = non-significant difference *p* ≥ 0.05; * *p* < 0.05; ** *p* < 0.01. Within each column, the means followed by the same letter indicate no significant differences at *p* = 0.05 (Tukey test).

## Data Availability

Data is contained within the article.
